# Biochemical and Epigenetic Regulation of Glutamate Metabolism in Maize (*Zea mays* L.) Leaves under Salt Stress

**DOI:** 10.3390/plants13182651

**Published:** 2024-09-21

**Authors:** Alexander T. Eprintsev, Galina B. Anokhina, Polina S. Selivanova, Polina P. Moskvina, Abir U. Igamberdiev

**Affiliations:** 1 Department of Biochemistry and Cell Physiology, Voronezh State University, Voronezh 394018, Russia; bc366@bio.vsu.ru (A.T.E.); dowi2009@mail.ru (G.B.A.); lol221297@mail.ru (P.S.S.); polinamoskvina2001@gmail.com (P.P.M.); 2 Department of Biology, Memorial University of Newfoundland, St. John’s, NL A1C 5S7, Canada

**Keywords:** glutamate dehydrogenase, mitochondria, NaCl, promoter methylation, 2-oxoglutarate dehydrogenase, salt stress, tricarboxylic acid cycle, *Zea mays* L.

## Abstract

The effect of salt stress (150 mM NaCl) on the expression of genes, methylation of their promoters, and enzymatic activity of glutamate dehydrogenase (GDH), glutamate decarboxylase (GAD), and the 2-oxoglutarate (2-OG)–dehydrogenase (2-OGDH) complex was studied in maize (*Zea mays* L.). GDH activity increased continuously under salt stress, being 3-fold higher after 24 h. This was accompanied by the appearance of a second isoform with lower electrophoretic mobility. The expression of the *Gdh1* gene strongly increased after 6–12 h of incubation, which corresponded to the demethylation of its promoter, while *Gdh2* gene expression slightly increased after 2–6 h and then decreased. GAD activity gradually increased in the first 12 h, and then returned to the control level. This corresponded to the increase of *Gad* expression and its demethylation. Salt stress led to a 2-fold increase in the activity of 2-OGDH during the first 6 h of NaCl treatment, then the activity returned to the control level. Expression of the genes *Ogdh1* and *Ogdh3* peaked after 1–2 h of incubation. After 6–8 h with NaCl, the expression of these genes declined below the control levels, which correlated with the higher methylation of their promoters. We conclude that salt stress causes a redirection of the 2-OG flux to the γ-aminobutyric acid shunt via its amination to glutamate, by altering the expression of the *Gdh1* and *Gdh2* genes, which likely promotes the assembly of the native GDH molecule having a different subunit composition and greater affinity for 2-OG.

## 1. Introduction

Salinity affects vast areas of agricultural lands, and the most common factor causing salt stress is NaCl [1]. The salinity tolerance mechanisms in plants include limiting ion uptake and compartmentalizing ions, which leads to the prevention of negative osmotic effects on cells. The role of mitochondrial metabolism in the adaptation to stress conditions is essential for developing a salt tolerance via an increase in ATP generation, the scavenging of reactive oxygen species (ROS), and the regulation of ion transport across membranes [2]. The increase in the oxidation of mitochondrial substrates is an important characteristic of the salt stress response usually referred as a “salt respiration” [3]. It is caused by stress-induced changes in the modulation of redox balance in mitochondria resulting in the activation of respiratory enzymes and transporters at the genetic level, epigenetic level, and at the level of posttranscriptional modification of proteins [4,5,6,7].

The effect of salt stress includes two distinct phases, the early osmotic phase and the later ionic phase. The first phase refers to the rapid onset of osmotic stress within minutes due to the fast decrease in extracellular water potential, and the second phase refers to ionic and oxidative stress, as well as nutrient imbalance resulting from the accumulation of ions (Na^+^ and Cl^−^ in the case of NaCl stress) over time. The consequences of the second phase of stress can be observed over several days [8,9,10,11]. Salt stress affects the entire plant organism, including at the molecular genetic level. This occurs via specific signaling mechanisms that provide a cellular response to stress factors. Plants adapt to salt stress through the synthesis of osmolytes such as proline and soluble sugars and amines that possess the osmoprotective activity [12,13,14]. Proline biosynthesis occurs via the glutamate and ornithine pathways, and, in addition to reducing cytosolic osmotic potential, it is important for stabilizing protein structures, scavenging free radicals, and maintaining intracellular ionic homeostasis [14].

The tricarboxylic acid (TCA) cycle undergoes significant changes as a result of salt stress. Mitochondrial respiration and the active operation of the TCA cycle are important sources of ATP in stress conditions. Under the conditions of salinity, in the first six hours of stress, the intensification of the TCA cycle takes place [15,16,17]. The increase in the TCA cycle enzymes, including the 2-oxoglutarate–dehydrogenase (2-OGDH) complex [18], as well as the succinate dehydrogenase, which directly supplies electrons to the electron transport chains of mitochondria [17], is due to the need for an additional influx of energy to neutralize the negative effects of salts on cellular metabolic processes. The 2-OGDH complex (EC 1.2.1.105) is a complex multienzyme system that provides oxidation of 2-oxoglutarate (2-OG) to succinyl-CoA in mitochondria while simultaneously reducing NAD^+^ to NADH. The 2-OGDH complex plays an important role in plant metabolism, catalyzing, firstly, the rate-limiting stage of mitochondrial respiration, and secondly, being a key participant in carbon–nitrogen interactions [19,20]. According to Che-Othman et al. [21], longer-term salt stress exhibits an inactivating effect on the activity of the 2-OGDH complex, which suggests that the operation of the TCA cycle is suppressed under these conditions. During the exposure to salinity, key metabolic enzymes necessary for the cyclic operation of the TCA cycle are inhibited by sodium chloride, which is overcome by the inclusion of an alternative pathway—the γ-aminobutyric acid (GABA) shunt—which provides an additional source of succinate for mitochondria [21,22]. This shunt bypasses the 2-OGDH complex via the decarboxylation of glutamate, which can be an alternative product of the 2-oxoglutarate (2-OG) conversion.

The connection between the TCA cycle and the compensatory stress response pathway, the GABA shunt, is ensured by the operation of GDH (EC 1.4.1.3), which catalyzes the reversible deamination of L-glutamate to 2-OG [23,24]. In addition to transamination, 2-OG can be converted to glutamate via the reverse reaction of glutamate dehydrogenase (GDH) [25,26]. In this reaction, the ammonium ion (NH_4_^+^) is utilized at a high concentration and at a high redox level [27]. These conditions are common in the case of salt stress [28]. GDH in plants is a simultaneous participant in both energy and biosynthetic metabolism, which is especially important from the point of view of cell adaptation to stress. A peculiarity of GDH in plants is that the catalytically active enzyme is formed via the assembly of a homo- or hetero-hexameric molecule consisting of α and/or β subunits. The predominance of α-subunits in the enzyme structure provides a higher affinity for glutamate, while the presence of β-subunits increases the affinity for 2-oxoglutarate [29,30,31].

The studies on cucumber leaves [13] showed that the activities of GDH (both in the amination and deamination directions), alanine aminotransferase, aspartate aminotransferase, and NADH-dependent isocitrate dehydrogenase changed after a prolonged (more than 24 h) exposure to salt stress. The activities of the above enzymes increased significantly throughout the experiment, which was associated with a high need for glutamate [13]. GDH induction at the genetic level in barley in the conditions of salt stress was demonstrated [32]. It is known that a high NaCl concentration stimulates the formation of reactive oxygen species (ROS), which in turn induce the synthesis of the α-subunit of GDH in tobacco and grapevines [33]. The important role of GDH in the adaptive response of plant cells to salt stress is confirmed by the report of Kumar et al. [34], which showed that in salt-tolerant varieties of rice (*Oryza sativa*), the activity of GDH in the direction of amination increases with the increasing salt stress, while in salt-sensitive varieties it decreases [34]. It has been shown that in pea (*Pisum sativum*) plants, which are resistant to ammonium excess, the aminating activity of GDH in roots is very high [35].

The glutamate formed during the amination of 2-oxoglutarate can be directed both to the synthesis of proline and to the formation of GABA, which in turn is a substrate for the GABA shunt, bypassing the 2-OGDH reaction. GDH isoforms that preferably operate in the direction of ammonia assimilation act as anti-stress enzymes in ammonia detoxification and glutamate production for proline synthesis [33]. In plants, GABA is formed primarily through the H^+^-consuming α-decarboxylation of glutamate in an irreversible reaction catalyzed by the cytosol-localized glutamate decarboxylase (GAD, EC 4.1.1.15), which occurs under low pH conditions with the participation of pyridoxal phosphate. GABA synthesized in the cytosol enters mitochondria via the mitochondrial GABA transporter and is converted first to succinic semialdehyde (SSA) by GABA transaminase, and then to succinate, which is part of the TCA cycle [36]. This last step greatly influences the redox status of the cell because succinate bypasses the three TCA cycle reactions that produce NADH [37,38].

The adaptation of plants to salt stress includes changes in the content of metabolites, which are regulated at the biochemical and the genetic levels. However, another option for regulating the metabolic flows of a plant cell during an adaptive response to stress is the involvement of epigenetic mechanisms, which include DNA methylation. The methylation of various regions of the genome of the facultative halophyte plant *Mesembryanthemum crystallinum* to soil salinity was demonstrated [39]. A unique feature of this plant is its ability to switch from the C_3_ pathway of photosynthesis to Crassulacean Acid Metabolism (CAM) under water deficiency and salinity. This switch is achieved through the activation of a large group of genes, among which the gene of the CAM-specific form of PEP carboxylase *Ppc1* is considered “diagnostic”. The transcription level of this gene increases sharply during adaptation to salt stress; nevertheless, no changes in the methylation pattern of its promoter region were detected, and the methylation of repetitive rRNA genes did not change either. However, total DNA methylation at CpNpG sites increased 2-fold during salt stress. It turned out that this increase was associated mainly, if not exclusively, with a satellite DNA fraction. Apparently, adaptation to salt stress is achieved due to the global epigenetic reorganization of chromatin, which ensures the modulation of the expression of a large group of genes [39].

Despite the extensive knowledge about possible adaptive mechanisms of cellular metabolism to the effects of salinity caused by NaCl, the mode of regulation and functioning of the 2-OGDH complex and GDH under short-term salinity stress remains unclear. In this regard, the goal of this study was to identify the regulatory aspects of the operation of the 2-OGDH complex and GDH in maize leaves in the first 24 h of salt stress caused by NaCl. We studied the expression and epigenetic regulation of the genes *ZmOgdh1* and *ZmOgdh3* encoding the E1 subunit of 2-OGDH, of the genes *ZmGdh1* and *ZmGdh2* encoding α and β subunits of GDH, and of the gene *ZmGad* encoding GAD (further the names of genes are given without *Zm* for brevity). Our study demonstrates that salt stress affects the expression of these genes, and that this process is partially regulated via epigenetic mechanisms.

## 2. Results

### 2.1. Activity of the 2-OGDH Complex

**Figure 1:**
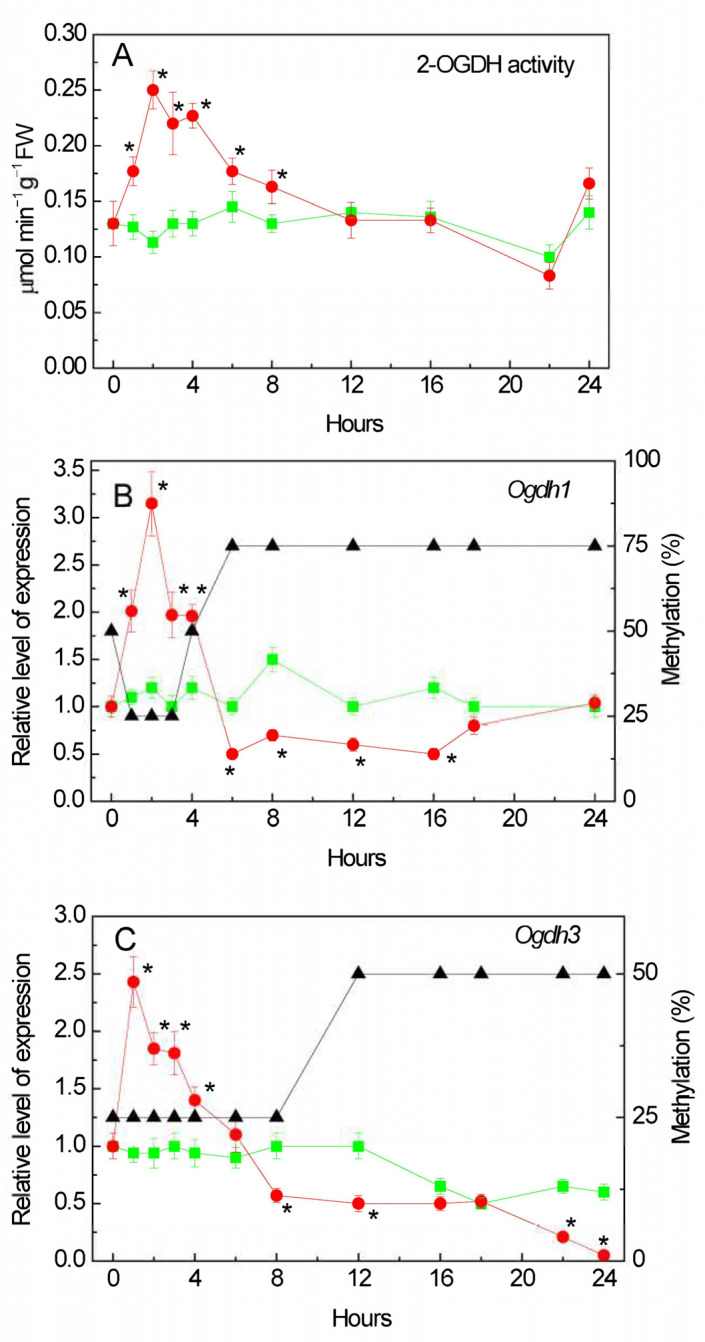
Effect of salt stress on the operation of the 2-oxoglutarate dehydrogenase (2-OGDH) complex. Changes in the activity of the 2-OGDH complex (**A**), in the relative levels of transcripts and the fraction of methylation of promoters (black triangles) of the genes *Ogdh1* (**B**) and *Ogdh3* (**C**) in maize leaves in the course of incubation of maize plants in 150 mM NaCl (red circles) as compared to the control plants (green squares). The data represent the means of three biological repeats ± SD. Statistically significant differences in activity and expression as compared to the control (*p* ≤ 0.05) are shown by stars.

The activity of the 2-OGDH complex increased in the first hour of incubation of the plants with the NaCl solution (Figure 1A). The maximum activity was more than twice that of the control plants at 2 h of incubation, and then it gradually decreased. After 12 h of incubation on the medium with NaCl, the activity became close to the control values.

### 2.2. Changes in 2-Ogdh Gene Expression

We studied the transcriptional activity of the two genes encoding the subunit E1 of the 2-OGDH complex in the maize genome: *Ogdh1* (LOC100383579) and *Ogdh3* (LOC100383847) encoding the E1 subunit (2-OG–dehydrogenase) of the 2-OGDH complex [40,41] (the date of access to the NCBI database was 26 September 2023). Salt stress stimulated the expression in both genes. In the first hours of the experiment, an increase in the level of *Ogdh1* gene transcripts was observed compared to the control samples (Figure 1B). The maximum concentration of the *Ogdh1* gene mRNA was recorded after 2 h of incubation of the plants in the NaCl solution. Further incubation of the plants in a NaCl solution led to a decrease in the expression of *Ogdh1*. The maximum effect of NaCl incubation on *Ogdh3* transcript level was achieved in the first hour of stress exposure (Figure 1C). An increased level of expression of the *Ogdh3* gene mRNA in the first hours of the experiment (up to 6 h) was followed by its gradual decline. After 24 h, the relative level of *Ogdh3* transcripts in the NaCl incubated plants was 20 times lower than in the control plants.

### 2.3. Changes in the Degree of Methylation of Individual CpG Dinucleotides in Ogdh Promoters

Changes in the mRNA level of the maize *Ogdh1* gene when the seedlings were exposed to NaCl correlated with the changes in the methyl status of individual CpG dinucleotides in the promoter. An increase in gene transcription was accompanied by a decrease in the amount of methylated cytosines, while a decrease in the gene mRNA concentration correlated with an increase in the proportion of methylated CpG dinucleotides (Figure 1B). At the same time, in the control samples, the degree of methylation was 50% throughout the entire experiment (Figure S1).

An analysis of the NaCl induced changes in the *Ogdh3* gene methylation and its expression showed that in the first hours of salt stress there was an induction of the functioning of this gene. Moreover, there were no fluctuations in the degree of methylation of individual CpG dinucleotides—the proportion of methylated cytosines during a 4 h exposure to NaCl was 25% (relative to the number of CpG dinucleotides studied) (Figure 1C). The decrease in the transcriptional activity of the *Ogdh3* gene observed at 12 h of the experiment is apparently associated with an increase in the number of methylated cytosines in the promoter region to 50% (relative to the CpG dinucleotides under study). In the control group of plants, the degree of methylation remained at a constant level and amounted to 25% (Figure S1).

### 2.4. Analysis of 2-Ogdh Gene Promoters for the Presence of CpG Islands

**Figure 2:**
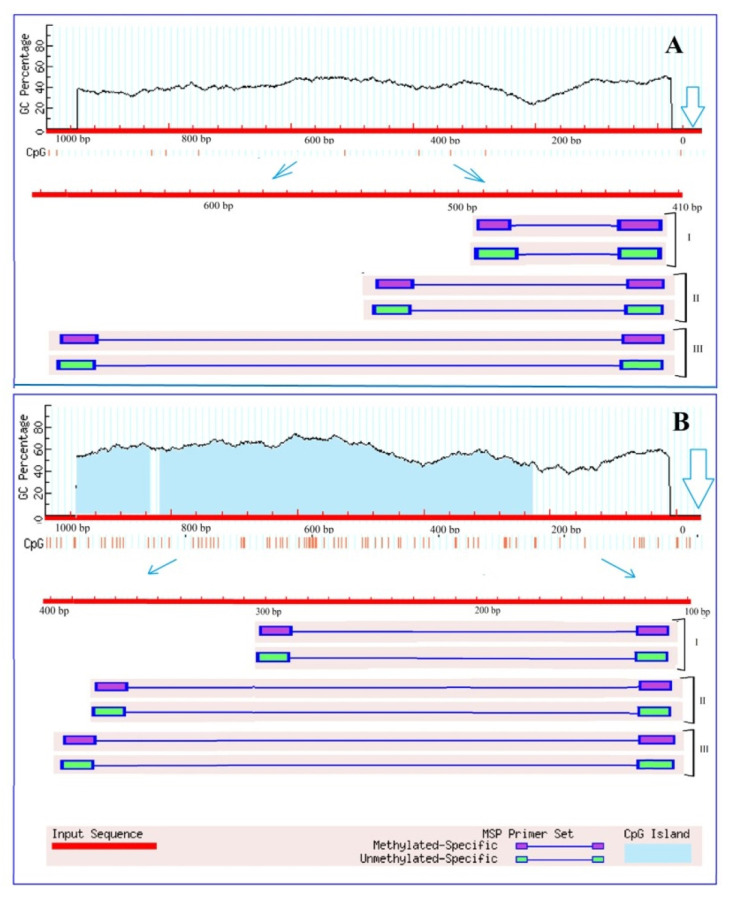
Results of analysis of the promoters of the genes *Ogdh1* (**A**) and *Ogdh3* (**B**) of *Zea mays* for the presence of CpG islands. Vertical lines indicate the positions of CpG dinucleotides. The outlined arrows indicate the position of the start codon. Thin blue arrows show the change of scale to outline the region used for designing three groups (I, II, III) of primers to the different CpG dinucleotides.

An analysis of the *Ogdh* gene promoters for the presence of CpG islands showed that there is no single CpG islands in the promoter region of the *Ogdh1* gene (Figure 2A), while the promoter region of *Ogdh3* contains two CpG islands with a size of 116 bp and 591 bp (Figure 2B).

Due to the fact that CpG islands are present in the promoter region of the gene, it can be assumed that the operation of this gene can be regulated through methylation. However, the absence of CpG islands does not exclude the possibility of controlling gene expression by changing the methyl status, since in plant organisms, unlike animals, cytosine methylation is possible not only at CpG sites, but also at CpNpG and CpNpN sites, where N is A, T, or C [42]. The analysis of the promoter region of the *Ogdh1* gene for the presence of CpNpG and CpNpN sites resulted in the estimation that the studied promoter region of 1000 bp in size contains 24.9% CpNpN sites and 11.4% CpNpG sites, which indicates the possibility of regulating gene transcription by changing the methyl status of its promoter.

### 2.5. Effect of Salt Stress on Glutamate Dehydrogenase Activity

**Figure 3:**
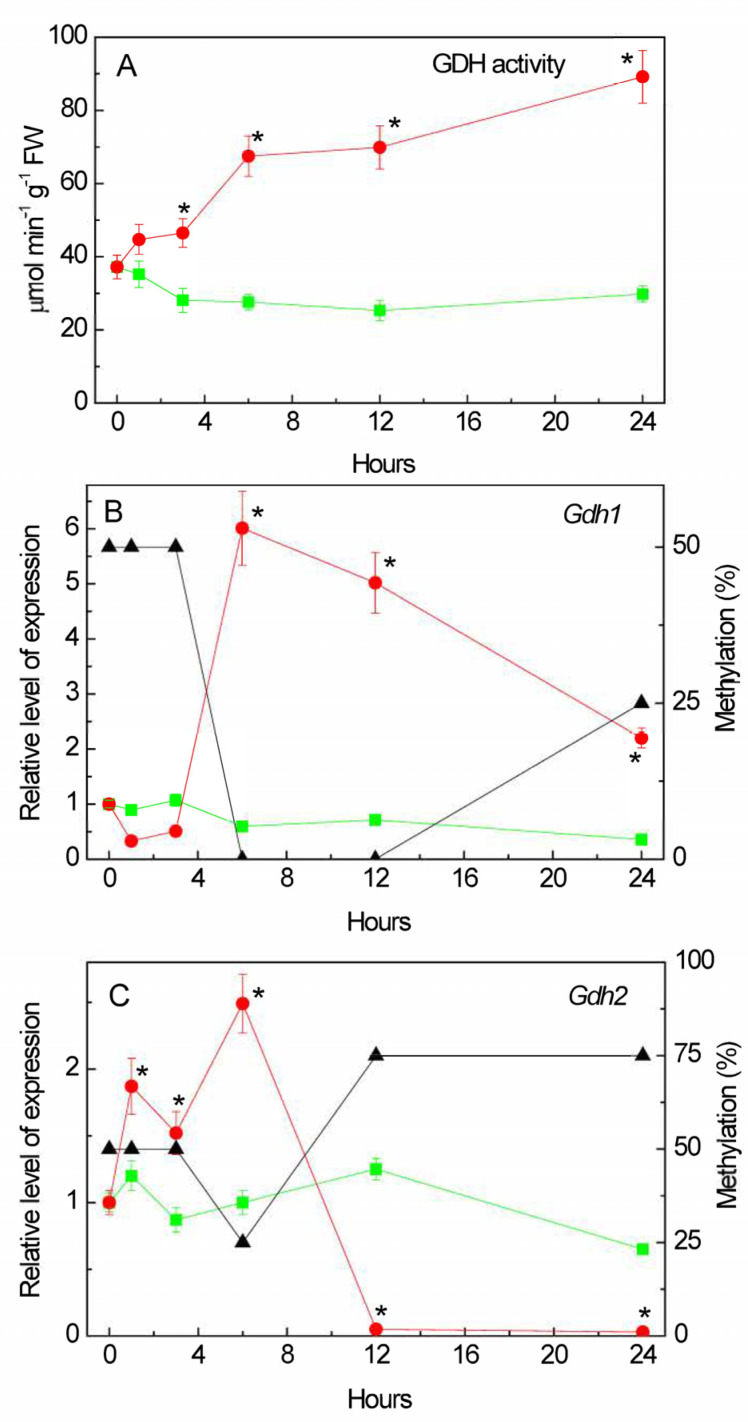
Effect of salt stress on glutamate dehydrogenase activity and expression in maize leaves. Changes in the activity of glutamate dehydrogenase (GDH) (**A**), in the relative levels of transcripts and the fraction of methylation of promoters (black triangles) of the genes *Gdh1* (**B**) and *Gdh2* (**C**) in maize leaves in the course of incubation of maize plants in 150 mM NaCl (red circles) as compared to the control plants (green squares). The data represent the means of three biological repeats ± SD. Statistically significant differences in activity and expression as compared to the control (*p* ≤ 0.05) are shown by stars.

Salinity induced the overall activity of GDH in the amination reaction (Figure 3A). An almost 1.5-fold increase in GDH activity was recorded after 1 h of NaCl incubation, then it further continuously increased, and at 24 h of incubation, the total GDH activity was three times higher than in the control.

### 2.6. Isoenzyme Composition of GDH under Salt Stress Conditions

**Figure 4:**
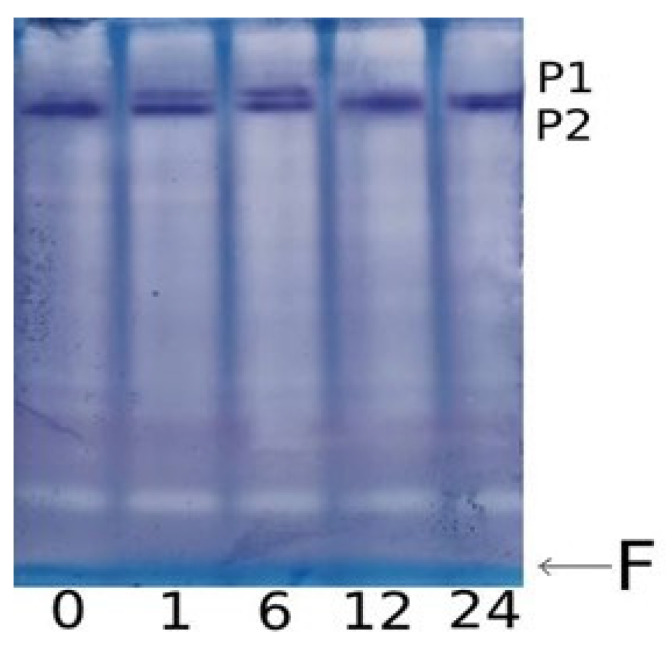
Effect of salt stress on the isoenzyme composition of GDH in maize leaves. PAGE electropherogram of GDH from maize leaves under salt stress: 0, 1, 6, 12, 24—incubation time in the NaCl solution (hours); P1, P2—protein bands representing native GDH protein molecules (isoenzymes) stained by the tetrazolium method; and F—dye front.

The increase in the overall enzymatic activity of GDH was accompanied by the appearance of an additional isoform with lower electrophoretic mobility on electropherograms (Figure 4). This second isoform was detectable after 1 h of incubation in the NaCl solution, and after 12 h of the experiment, it disappeared from electropherograms. Before the start of the experiment, as well as in the plants from the control group, only one form of GDH with an electrophoretic mobility value of 0.17 was observed; however, starting from the first hour of incubation in a saline solution, the appearance of a second isoform with a mobility value of 0.13 was noted. It is known that the α-subunit has greater electrophoretic mobility than the β-subunit [43], which suggests that the appearance of the second isoenzyme is due to the occurrence in the structure of the polypeptide of the less mobile β-subunit.

### 2.7. Changes in the Expression of Gdh Genes under Salt Stress

The induction of GDH activity under salt stress can be associated with changes in the functioning of the *Gdh1* (LOC542220) and *Gdh2* (LOC100193614) genes [44,45] (Figure 3B,C). The relative level of *Gdh1* gene transcripts, despite the initial reduction in the first 5 h of the experiment, increased at 6 h of salt stress. The low level of expression of this gene in the first hours of salinity was compensated by the operation of the *Gdh2* gene, for which transcriptional activity increased after 1 h of incubation in the solution of NaCl (Figure 3C). This corresponds to the appearance of an isoform with lower electrophoretic mobility (Figure 4). Starting from 6 h, the expression of the *Gdh1* gene increased.

**Figure 5:**
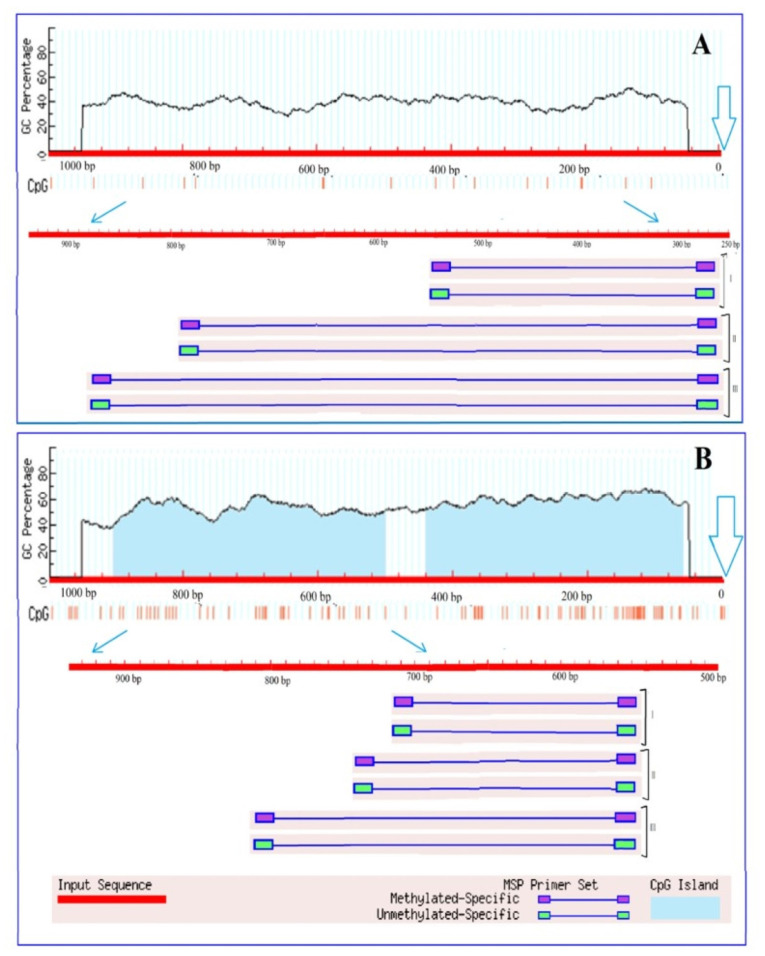
Results of analysis of the promoters of the genes *Gdh1* (**A**) and *Gdh2* (**B**) of *Zea mays* for the presence of CpG islands. Vertical lines indicate the positions of CpG dinucleotides. The outlined arrows indicate the position of the start codon. Thin blue arrows show the change of scale to outline the region used for designing three groups (I, II, III) of primers to the different CpG dinucleotides.

An analysis of the promoters of the *Gdh* family genes for the presence of CpG islands showed that the promoter of the *Gdh1* gene does not contain a single CpG island (Figure 5A), while two islands of 404 and 383 bp in size were found in the promoter region of the *Gdh2* gene (Figure 5B). A study of the promoter region of the *Gdh1* gene for the presence of CpNpG and CpNpN sites revealed that the structure of the 1000 bp promoter region contains 24.9% CpNpN sites and 13.8% CpNpG sites. A study of changes in the degree of methylation of individual CpG dinucleotides in the *Gdh1* gene promoter revealed that the increase in the mRNA concentration at 6 h of stress exposure was associated with a complete demethylation of the dinucleotides under study, which were 50% methylated before the experiment and in the first 3 h of the experiment (Figure 3B). An increase in the concentration of the *Gdh2* gene mRNA at 6 h of salt stress was accompanied by a decrease in the proportion of methylated cytosines (from 50 to 25%) in its promoter, and further inactivation of the gene corresponded to an increase in the degree of methylation to 75% (Figure 3C). In the control group of plants, the degree of methylation of the promoters of both genes was 50% throughout the entire duration of the experiment (Figure S2).

### 2.8. Effect of Salt Stress on Glutamate Decarboxylase Activity

**Figure 6:**
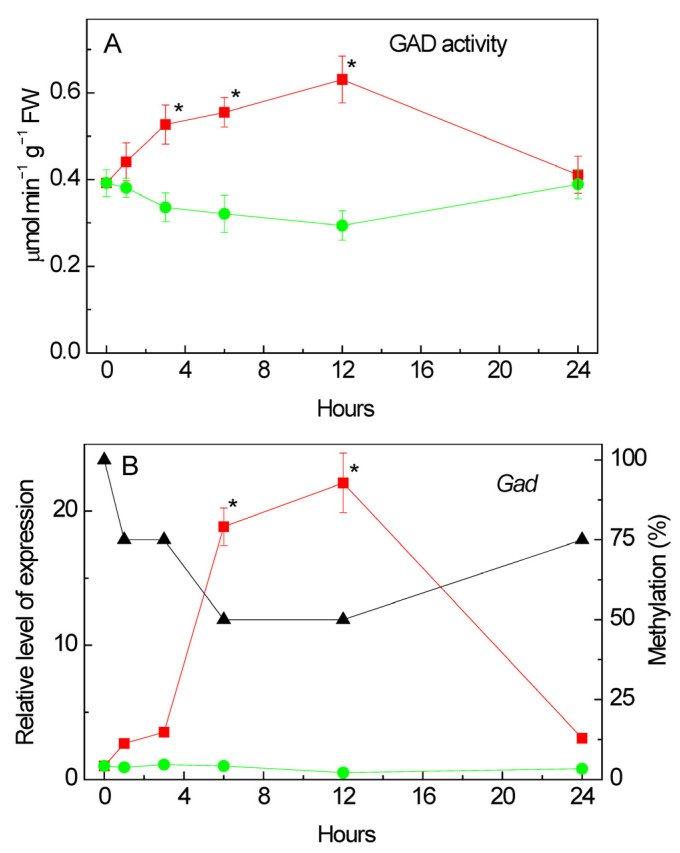
Effect of salt stress on glutamate decarboxylase (GAD) activity and expression in maize leaves. Changes in the total enzymatic activity of GAD (**A**), and in the relative levels of transcripts and the fraction of methylation of the promoters (black triangles) of the gene *Gad* (**B**) in maize leaves in the course of incubation of maize plants in 150 mM NaCl (red circles) as compared to the control plants (green squares). The data represent the means of three biological repeats ± SD. Statistically significant differences in activity and expression as compared to the control (*p* ≤ 0.05) are shown by stars.

An analysis of the total enzymatic activity of glutamate decarboxylase (GAD) in corn leaves under salinity conditions caused by 150 mM of sodium chloride showed that from the first hour of stress exposure there already was a gradual increase in the studied parameter relative to the values recorded in the control group of plants (Figure 6A). The maximum increase in GAD activity was observed at 12 h of the experiment. In the following hours, a gradual decrease in the values of the catalytic activity of GAD was recorded.

### 2.9. Changes in the Expression of Gad under Salt Stress

**Figure 7:**
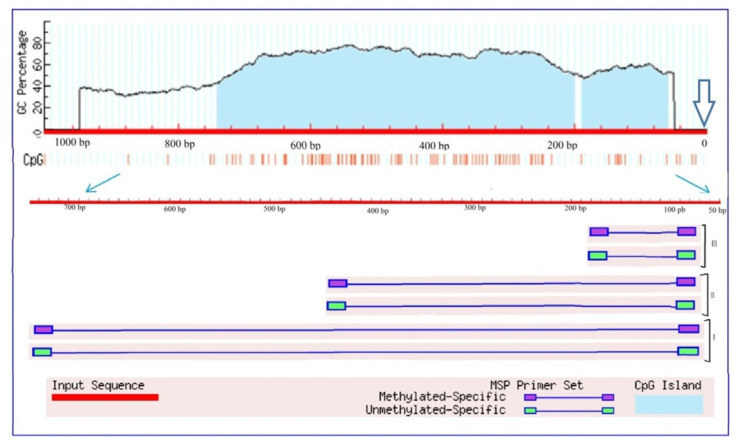
Analysis of *Gad* gene promoters of *Zea mays* for the presence of CpG islands. Vertical lines indicate the positions of CpG dinucleotides. The outlined arrow indicates the position of the start codon. Thin blue arrows show the change of scale to outline the region used for designing three groups (I, II, III) of primers to the different CpG dinucleotides.

An analysis of the transcriptional activity of the *Gad* gene (LOC100284394) [46], which encodes glutamate decarboxylase in the maize genome, showed an increase in the relative level of transcripts from the first hour of stress exposure (Figure 6B). The maximum increase in the mRNA level was observed at 12 h of the experiment and exceeded the control values by more than 20 times. By 24 h of the experiment, a significant decrease in the transcriptional activity of the *Gad* gene was noted; however, the relative level of transcripts in the group exposed to salt stress was more than three times higher than in the control group of plants. An analysis of the nucleotide sequence of the promoter region of the *Gad* gene showed the presence in its structure of two CpG islands measuring 130 and 543 bp (Figure 7).

A comparative analysis of the changes in the relative level of gene transcripts and the degree of methylation of its promoter showed that an increase in the transcriptional activity of the *Gad* gene is accompanied by a decrease in the degree of promoter methylation to 50%. Before the start of the experiment, all dinucleotides under study were methylated; however, by the 6th hour of the experiment, the degree of methylation of the individual CpG dinucleotides promoter was 50%. The decrease in the mRNA level at 24 h of the experiment was associated with an increase in the methyl status of the promoter region of the *Gad* gene. Thus, changes in the transcriptional activity of the *Gad* gene are likely due to changes in the methyl status of its promoter, which indicates the epigenetic nature of the regulation of this gene in maize leaves under salinity conditions caused by 150 mM of sodium chloride. In the control group of plants, 50% of all studied CpG dinucleotides were methylated throughout the entire experiment (Figure S3).

## 3. Discussion

The obtained data reveal significant changes in the activity and expression of the enzymes involved in 2-OG conversion under the conditions of salt stress. The 2-OGDH multienzyme complex catalyzes the rate-limiting stage of the TCA cycle [18]. The incubation of maize seedlings in the NaCl solution in the first six hours leads to the intensification of the TCA cycle due to an increase in the activity of the 2-OGDH complex in leaves. The operation of this complex is regulated at the biochemical [20], genetic [47,48], and epigenetic (in this study) levels. An additional influx of 2-OG to the TCA cycle due to an increase in the deamination activity of GDH leads to further intensification of the formation of succinyl-CoA. This is evidenced by the appearance of an additional GDH isoenzyme, which is caused by an increase in the expression profile of the *Gdh2* gene. These assumptions are confirmed by data obtained from Tercé-Laforgue et al. [43], who showed that a polypeptide containing in its structure the β-subunit encoded by the *Gdh2* gene has lower electrophoretic mobility during PAGE electrophoresis than the GDH enzyme consisting of α-subunits. GDH, consisting predominantly of β-subunits, has high deamination activity, thus the direction of the reaction is shifted towards the formation of 2-OG, while the polypeptide consisting of α-subunits is characterized by a high affinity for 2-OG, which contributes to the redirection of the reaction towards the formation of glutamate in the amination reaction [43].

The initial rapid induction of the TCA cycle under conditions of salt stress, caused by an increase in the catalytic activity of its enzymes in the first hours of incubation, is known as “salt respiration” [6,17]. The observed increase in the activity of the 2-OGDH complex in the first hours of salinity indicates an increase in the rate of functioning of the entire TCA cycle. Since the 2-OGDH complex provides the oxidation of 2-OG through the interaction of its three components, while the slowest reaction in this complex multi-step process is catalyzed by 2-OG dehydrogenase (E1 subunit; E.C. 1.2.4.2), a study was carried out on the dynamics of the transcriptional activity of genes encoding the subunit E1 (*Ogdh1* and *Ogdh3*).

A study of the mRNA level of genes encoding 2-OGDH showed that salt stress stimulates their expression: in the first hours of the experiment, an increase in the level of *Ogdh1* gene transcripts was observed compared to the control samples (Figure 1A,B). Further incubation of the seedlings in a NaCl solution led to a decrease in the expression of this gene, which corresponded to the decline in the enzymatic activity of the 2-OGDH complex compared to the control level. The level of *Ogdh3* gene transcripts increased even earlier, and it was also followed by a gradual decline in its transcriptional activity (Figure 1C). The decline in the relative level of *Ogdh3* transcripts in maize leaves after 24 h of salt stress was substantial, being ~20 times lower than in the leaves of the control group; thus, the activity of 2-OGDH in the conditions of salt stress was supported by the *Ogdh1* gene.

There is compelling evidence that epigenetic modifications may be an important mechanism of adaptation to different habitats. Moreover, there is a large amount of data indicating changes in the degree of methylation of both the entire genome and its individual regions are induced by stress [49]. The transcriptional activity of genes can be regulated by changing the methyl status of both individual CpG dinucleotides and CpG dinucleotides within the CpG island [50]. In addition, it is known that DNA methylation regulates the activity of enhancers through changes in the methyl status of the transcription factor binding sites [49]. An analysis of the OGDH gene promoters for the presence of CpG islands showed that there is not a single CpG island in the promoter region of the *Ogdh1* gene (Figure 2A), while the promoter region of *Ogdh3* contains two CpG islands (Figure 2B). The presence of CpG islands in the promoter region suggests that the operation of the corresponding gene is regulated through methylation. However, the absence of CpG islands does not exclude the possibility of controlling gene expression by changing the methyl status, since in plant organisms, unlike animals, cytosine methylation is possible not only at CpG sites, but also at CpNpG and CpNpN sites [51]. Changes in the methyl status of the CpG dinucleotides of the *Ogdh1* and *Ogdh3* gene promoters, correlating with the transformation of the relative level of their transcripts, indicate the important role of DNA methylation in the regulation of the operation of the 2-OGDH complex during the adaptive response of cellular metabolism to salinity conditions.

The disappearance of the effect of salt respiration at the level of the 2-OGDH complex during the long-term (more than 6 h) incubation of seedlings in a 150 mM NaCl solution (Figure 1) indicates a possibility of a switch in the 2-OG flow in the mitochondria using the reverse reaction of GDH by directing it to the GABA shunt [17]. This redistribution of the key metabolite of the TCA cycle is due to the fact that the 2-OGDH activity is reduced after 6 h of salt stress, which corresponds to an increase in the proportion of methylated cytosines in the promoters of the *Ogdh1* and *Ogdh3* genes (Figure 1B,C). If 2-OGDH does not utilize all accumulated 2-OG, this may lead to the activation of a GDH isoform with a higher capacity in the amination reaction.

The induction of GDH activity under salt stress is associated with the changes in the functioning of the *Gdh1* and *Gdh2* genes (Figure 3). In the *Zea mays* L. genome, GDH is encoded by two genes, each of which contains information about different types of GDH subunits, α and β [52]. The predominance of one or another type of subunit in the structure of the polypeptide leads to a shift in the equilibrium of the reaction towards the formation of 2-OG, or, conversely, towards the synthesis of glutamate. The activation of glutamate conversion to 2-OG corresponds with the appearance of an isoform with lower electrophoretic mobility (Figure 4), which possibly contains in its structure the α-subunit of GDH, providing the displacement of the reaction towards the formation of 2-OG, which subsequently enters the TCA cycle, to maintain energy metabolism. Then, starting from 6 h, the *Gdh1* gene is activated (Figure 3), encoding the β-subunit of GDH, which promotes the reaction towards the formation of glutamate, which can be used for the synthesis of proline [29,34,35] or decarboxylated to form GABA [22,37]. The observed changes in the expression of the *Gdh1* gene under salt stress conditions are associated with a transformation of the methyl status, indicating an epigenetic mechanism for the regulation of its transcription. An increase in the expression of the *Gdh1* gene, caused by a decrease in the degree of cytosine methylation of this gene, as well as an increase in the proportion of methylated cytosines in the promoter of the *Gdh2* gene, provides an alternative pathway for the conversion of 2-OG, which results in the activation of the GABA shunt.

A change in the expression of the GDH genes “switches” the flow of 2-OG from the TCA cycle to amino acid synthesis. It should be noted that GDH activity both in the first 6 h and at the second stage of stress exposure (after 6 h) was regulated due to the differential expression of the *Gdh1* and *Gdh2* genes. At the same time, the processes of methylation/demethylation of CpG dinucleotides within their promoters play a key role in the regulation of the GDH activity (Figure 3 and Figure 5). Glutamate entering the GABA shunt is decarboxylated to GABA, which is converted via succinic semialdehyde to succinate, and the latter subsequently enters the TCA cycle in the consequent reaction. This bypasses the limiting step of the TCA cycle at the level of the 2-OGDH complex and provides efficient adaptation to the conditions of salt stress.

Thus, the incubation of maize seedlings in a NaCl solution results in a long-term induction of GDH, which includes a short-term induction of the second isoform. Both genes (*Gdh1* and *Gdh2*) participate in the regulation of the GDH activity under salt stress. The appearance of an additional isoform in the first hours of the experiment is associated with the formation of a form of GDH consisting of α-subunits, the predominance of which in the structure of the polypeptide ensures the occurrence of an enzymatic reaction towards the formation of 2-OG, and contributes to the maintenance of the TCA cycle during the short period of “salt respiration”. Long-term exposure of maize seedlings to NaCl (6 h or more) induces the transcription of the *Gdh1* gene mRNA, resulting in the synthesis of β-subunits of the enzyme. This leads the 2-OG from the TCA cycle to its amination, thereby turning it into glutamate, which can then be used for the synthesis of proline and GABA, which is confirmed by the data on changes in the expression and activity of GAD (Figure 6). The regulation of the glutamate dehydrogenase genes *Gdh1* and *Gdh2* under salt stress was carried out epigenetically due to changes in the methyl status of the CpG dinucleotides in their promoters.

**Figure 8:**
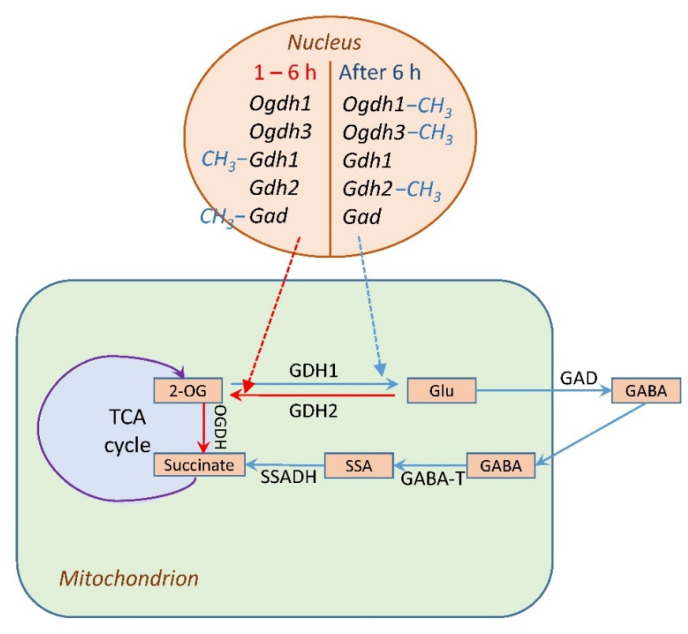
Regulation of glutamate metabolism in plant cells under salt stress. Abbreviations: TCA cycle, tricarboxylic acid cycle; 2-OG, 2-oxoglutarate; OGDH, 2-oxoglutarate dehydrogenase complex; GDH1 and GDH2, polypeptides encoded by the *Gdh1* and *Gdh2* genes, respectively; Glu, glutamate; GABA, γ-aminobutyric acid; SSA, succinic acid semialdehyde; Suc, succinate. Salt stress affects plant cell metabolism in two stages: 1. During the first 6 h of salt stress, 2-OGDH activity increases, while GDH, due to the induction of *Gdh2* gene expression, acts as a supplier of 2-OG, the source of which is glutamate (red arrow). 2. After 6 h, 2-OGDH is inhibited, GDH redirects the flow of 2-OG to glutamate due to the induction of the *Gdh1* gene, 2-OG is converted into glutamate, which is used for the synthesis of GABA (blue arrow). −CH_3_ indicates the methylation of gene promoter.

The initial activation of expression of the subunit E1 of the 2-OGDH complex provides the conversion of an additional pool of 2-OG formed from glutamate. After 6 h of salt stress, the limitations in 2-OG conversion would result in its amination to glutamate, which is directed to proline and GABA (Figure 8). A possibility of metabolite channeling in the TCA cycle [53] can facilitate these conversions and result in an efficient rearrangement of the mitochondrial metabolism for its adjustment to the conditions of salt stress.

## 4. Material and Methods

### 4.1. Object of Investigation

The object of research was two-weeks-old maize seedlings (*Zea mays* L., cv Voronezhskaya-76, obtained from the Voronezh branch of the All-Russian Research Institute of Maize), grown hydroponically under 10 h of daylight with a light intensity of 90 µmol quanta m^−2^ s^−1^ (climatic chamber “LabTech”, Namyangju, Republic of Korea) and an ambient temperature of 25 °C.

### 4.2. Induction of Salt Stress

The induction of salt stress was achieved by placing the plants, with the roots removed, in a 0.15 M NaCl solution for 24 h. Plants placed in water for the duration of the experiment were used as a control.

### 4.3. Determination of 2-OGDH, GDH and GAD Activities

#### 4.3.1. Extraction of 2-OGDH and GDH

The activities of 2-OGDH and GDH were determined in the mitochondrial fraction. For this purpose, the plant material was homogenized in the grinding medium containing 0.15 M potassium phosphate buffer (pH 7.4), 0.4 M sucrose, 2.5 mM EDTA, 1 mM KCl, and 4 mM MgCl_2_ in a ratio of 1 g plant material to 10 mL grinding medium. The homogenate was filtered through four layers of cheesecloth and centrifuged for 3 min at 3000× *g*. The supernatant was centrifuged for 10 min at 20,000× *g*. The pellet was resuspended in 50 mM potassium phosphate buffer (pH 7.4). Thiamine pyrophosphate (final concentration 0.2 mM) was added to the mitochondrial suspension when resuspending the sediment to measure 2-OGDH and GDH activity. The degree of mitochondrial breakage was more than 90%, which was controlled by microscopy on an Olympus CX41RF (“Olympus”, Tokyo, Japan). The cross-contamination of mitochondria by the cytoplasmic fraction did not exceed 5%, by the chloroplast fraction was 10%, and by the peroxisomal fraction was 3%; this was determined by measuring the enzymes specific to each fraction as described earlier [54].

#### 4.3.2. Extraction of GAD

The extraction of GAD was carried out by homogenizing the plant material in a 20 mM acetate buffer (pH 4.8) with the addition of 10 mM of pyridoxal phosphate, after which it was centrifuged at 12,000× *g* for 30 min. The supernatant was used for the GAD activity assay. All procedures were carried out at 4 °C.

#### 4.3.3. Determination of 2-OGDH Activity

2-Oxoglutarate–dehydrogenase complex (2-OGDH; EC 1.2.1.105) activity was determined in the mitochondrial fraction at 25 °C on an Evolution 260 Bio spectrophotometer (Thermo Fisher Scientific, Waltham, MA, USA) by the rate of NADH formation at 340 nm in a 0.1 M Tris-HCl buffer (pH 7.5) containing 1 mM potassium 2-oxoglutarate, 2 mM NAD^+^, 0.5 mM MgCl_2_, 0.12 mM CoA (lithium salt), 0.2 mM TPP, 2.5 mM cysteine, 1 mM AMP, and 0.05% Triton X-100 [18].

#### 4.3.4. Determination of GDH Activity

For the L-Glutamate, NAD(P)-oxidoreductase (GDH; EC 1.4.1.3) activity in the amination reaction was determined at 340 nm in 0.1 M Tris-HCl buffer (pH 8.0) containing 13 mM 2-oxoglutarate, 0.25 mM NADH, 1 mM CaCl_2_, and 50 mM (NH_4_)_2_SO_4_ on an Evolution 260 Bio spectrophotometer (Thermo Fisher Scientific, Waltham, MA, USA) [55].

#### 4.3.5. Determination of GAD Activity

The determination of glutamate decarboxylase (GAD; EC 4.1.1.15) activity was carried out using an Evolution 260 Bio spectrophotometer (Thermo Fisher Scientific, Waltham, MA, USA) by measuring changes in optical density at 620 nm for a solution containing a 20 mM acetate buffer (pH 4.8), 70 μM bromocresol green, 10 mM pyridoxal-5-phosphate, and 2 mM sodium glutamic acid [56]. The method is based on recording changes in the pH of the medium in the course of the reaction, which is reflected in the appearance of the protonated form of bromocresol green. The protonated form of bromocresol green has an absorption maximum at the wavelength of 620 nm.

One unit of enzymatic activity of GDH, GAD, and 2-OGDH corresponds to the conversion of 1 μmol of the substrate per minute at 25 °C and the optimal pH value.

#### 4.3.6. Electrophoretic Separation of GDH Isozymes

The isoenzyme patterns of GDH were determined by PAGE electrophoresis at 2–4 °C, according to Maurer [57]. The concentration of acrylamide in the upper gel was 4%, and in the lower gel was 7%. Equal amounts of protein (determined by the Lowry method [58]) were added to each pocket of the gel. The specific development of GDH was carried out using the tetrazolium method by incubating the gel in the dark in a 0.1 M Tris-HCl buffer (pH 8.0) containing 1 mM phenazine methosulfate (PMS), 1.8 mM nitroblue tetrazolium (NBT), 2 mM NAD^+^, and 10 mM sodium glutamate [59].

### 4.4. Expression of Gdh, Gad, and Ogdh Genes

To assess changes in the transcriptional activity of the *Gdh*, *Gad*, and *Ogdh* genes in maize leaves, we analyzed changes in the relative levels of transcripts of the *Gdh1* (*LOC542220*) and *Gdh2* (*LOC100193614*) genes encoding the α and β subunits of GDH, the *Gad* (*LOC 100284394*) gene encoding GAD, and the *Ogdh1* (*LOC100383579*) and *Ogdh3* (*LOC100383847*) genes encoding the subunit E1 of 2-OGDH in real-time PCR using specific primers (Tables S1–S3). The efficiency of the primers was estimated in the range of 0.2–0.8 μM by performing a series of dilutions followed by amplification by real-time PCR. The range of primer efficiency for the genes *Gdh1*, *Gdh2*, *Gad*, *Ogdh1*, and *Ogdh3* was 0.3–0.5 μM.

The progress in sequencing of the maize genome [60,61] made it possible to identify the genes encoding the studied enzymes. As a template for RT-PCR, we used cDNA obtained through a reverse transcription reaction with the MMLV-RT Kit (JSC Evrogen, Moscow, Russia) in accordance with the manufacturer’s protocol. RNA for the reverse transcription reaction was obtained through a phenol–chloroform extraction using LiCl to remove DNA [62,63]. The plant material (100 mg tissue per 1 mL medium) was ground in a porcelain mortar with an extraction medium containing 4 M guanidine thiocyanate, 30 mM sodium citrate, 30 mM β-mercaptoethanol, and a pH between 7.0 and 7.5; then 2 M of sodium acetate (pH 4.0) was added (1/10 of the volume) and mixed, followed by adding 1 volume of phenol saturated with water. After vortexing, a mixture of chloroform and isoamyl alcohol (49:1) was added, mixed (20 s), and incubated on ice for 15–20 min. After centrifugation for 20 min at 10,000*× g* at 4 °C, the upper aqueous phase was transferred to a new Eppendorf tube and 1 mL of isopropanol was added, after which the mixture was incubated at −20 °C for 1 h. The RNA was precipitated by centrifugation for 20 min at 10,000*× g* at 4 °C. The supernatant was removed, and the pellet was washed twice with 500 μL of 80% ethanol, dried and dissolved in RNase-free water (100 μL).

To purify the RNA from DNA contamination, 1 volume of 12 M lithium chloride was added, after which the sample was incubated at −20 °C for 30 min and centrifuged at 10,000*× g* for 15 min. The supernatant containing DNA was removed, and the RNA sediment was washed twice with 80% ethanol, then dried and dissolved in 40 µL of RNase-free water.

A real-time PCR was carried out on a LightCycler96 device (Roche, Solna, Sweden), using SYBR Green I as an intercalating dye. Amplification was carried out according to the following parameters: initial denaturation at 95 °C, for 5 min; then 35 cycles, including the stages of denaturation at 95 °C for 20 s, and at 72 °C for 20 s. Finally, a 10 min final elongation was performed at 72 °C. Quantitative matrix control was performed using gene-specific primers for housekeeping genes (Ef-1α elongation factors) (Table S4). Total RNA without the RT-PCR step was used as a negative control. Calculation of the relative levels of transcripts of the studied genes was carried out using the 2^−∆∆Ct^ method [64].

### 4.5. Analysis of the Promoters of the Studied Genes for the Presence of CpG Islands

An analysis of the promoters of the studied genes for the presence of CpG islands and selection of primers for methyl-specific PCR (Tables S5–S7) were carried out using the MethPrimer online service. The choice of the region for which the primers were selected was determined by the presence of individual CpG dinucleotides, as well as of plant-specific methylation sites (CNN, CNG). The analysis of potential targets was performed manually considering the microenvironment of the individual CpG dinucleotides, the presence of promoter regulatory elements near the studied CpG dinucleotides, and the methylation/demethylation of CpG dinucleotides (as well as CNN and CNG nucleotides) near the start codon.

### 4.6. Analysis of the Degree of Methylation of CpG Dinucleotides by Methyl-Specific PCR

To study the methyl status of individual CpG dinucleotides in the promoters of the genes under study, methyl-specific PCR was performed using ScreenMix (JSC Evrogen, Moscow, Russia), using DNA modified with sodium bisulfite as a template with some modifications [65]. In the first stage, pre-denatured DNA (incubation at 55 °C in the presence of 0.3 M NaOH) was modified by adding 500 µL of a mixture of 2.3 M NaHSO_3_ solution (Sigma-Aldrich, St. Louis, MO, USA) and 0.5 M hydroquinone (Sigma-Aldrich, St. Louis, MO, USA), followed by incubation in the dark at 55 °C for 4 h. At the second stage, the modified DNA was purified from sodium bisulfite using the DNeasy Plant Pro Kit (Qiagen, Hilden, Germany).

Desulfonation of the modified DNA was carried out by incubation with 0.3 M of NaOH (final concentration) for 20 min at 37 °C. To precipitate DNA from the solution, 2 μL of glycogen solution (20 mg/mL), 35 μL of 10 M ammonium acetate, and 3 volumes of 80% ethanol were added followed by incubation for 20 min at −20 °C. Then, it was centrifuged for 30 min at 13,000*× g*, washed twice with cooled 80% ethanol, and dried and dissolved in DNase-free water.

Amplification was carried out on a Mini AMP Thermal cycler (Thermo Fisher, Waltham, MA, USA) according to the following scheme: initial denaturation (10 min, 95 °C), 35 cycles of amplification, and final elongation (10 min, 72 °C). The cycles of amplification included denaturation (30 s, 95 °C), amplification (30 s, 52 °C), and elongation (30 s, 72 °C).

A calculation of the percent of methylation of the promoters of the studied genes was performed based on the results of the electrophoresis of the methyl-specific PCR amplicons. A visualization of the PCR products was carried out in a 2% agarose gel (Helicon, Moscow, Russia) with the addition of ethidium bromide (Sigma-Aldrich, St. Louis, MO, USA) [66]. For visualization, a Serva Blue cube 300 transilluminator (SERVA Electrophoresis GmbH, Heidelberg, Germany) with a wavelength of 312 nm was used. The degree of methylation of the promoter region is the total value of the results of the methyl-specific PCR of the studied CpG dinucleotides of a particular gene. To assess the degree of promoter methylation (the fraction of methylated CpG dinucleotides), the following criteria were used: 0% methylation—all three studied CpG dinucleotides were unmethylated; 25% methylation—1 or 2 CpG dinucleotides were partially methylated or 1 CpG dinucleotide was completely methylated; 50% methylation—1 or 2 CpG dinucleotides were completely methylated or all three examined CpG dinucleotides were partially methylated; 75% methylation—1 or 2 CpG dinucleotides were partially methylated, and the rest were fully methylated; and 100% methylation—all three CG dinucleotides were methylated [67]. This method provides an estimation of the degree of methylation in our experiments with a high precision (*p* < 0.01) within the frame of the four defined degrees of methylation.

### 4.7. Statistical Analysis

The experiments were performed in three biological replicates; analytical determinations for each sample were carried out in triplicate. The STATISTICA 12.0 program was used. The quantitative indicators were assessed for compliance with normal distribution using the Shapiro–Wilk test. The data distribution was assessed as normal distribution. The differences were analyzed for statistical significance using the Student’s *t*-test. The results in the graphs were expressed as mean ± SD. Additionally, a one-way analysis of variance ANOVA was used [68]. The differences discussed in this study are statistically significant (*p* ≤ 0.05).

## 5. Conclusions

In the first six hours (stage 1 of exposure) of salt stress, the TCA cycle is intensified due to an increase in the catalysis of the limiting reaction catalyzed by the 2-OGDH complex. The rate of operation of this metabolon is regulated at the biochemical, genetic, and epigenetic levels. Salt stress results in a short-term increase in the expression of the genes *Ogdh1* and *Ogdh3* encoding the E1 subunit of the 2-OGDH complex, due to a decrease in the proportion of methylated cytosines in the promoters of these genes. This provides the conversion of 2-OG formed from glutamate in the reaction of GDH. After 6 h of salt stress, the limitations in 2-OG conversion would result in its amination to glutamate, which is directed to GABA via GAD and proline. This rearrangement of the mitochondrial metabolism is important for the adjustment of plant cells to the conditions of salt stress. The process of coordinated changes in the activities of the 2-OGDH complex, GAD, and GDH is partially regulated via epigenetic mechanisms.

## Data Availability

The datasets generated for this study are available upon request from the corresponding author.

## Supplementary Materials

The following supporting information can be downloaded at: https://www.mdpi.com/article/10.3390/plants13182651/s1, Figure S1: Changes in the relative levels of transcripts of the genes *Ogdh1* and *Ogdh3* and of the fraction of methylation of their promoters in the control (non-stressed) maize plants.; Figure S2: Changes in the relative levels of transcripts of the genes *Gdh1* and *Gdh2* and of the fraction of methylation of their promoters in the control (non-stressed) maize plants; Figure S3: Changes in the relative levels of transcripts of the gene *Gad* and of the fraction of methylation of its promoters in the control (non-stressed) maize plants. Table S1: Primers to the genes encoding glutamate dehydrogenaseTable S2: Primers to the genes of glutamate decarboxylase ; Table S3: Primers to the genes of 2-oxoglutarate dehydrogenase; Table S4: Primers to the genes encoding Ef-1α; Table S5: Oligonucleotides for methyl-specific PCR for the primers of *Gdh1* and *Gdh2*; Table S6: Oligonucleotides for methyl-specific PCR for the primers of *Gad*; Table S7: Oligonucleotides for methyl-specific PCR for the primers of *Ogdh1* and *Ogdh3*.

## Abbreviations

GABA	γ-aminobutyric acid
GAD	glutamate decarboxylase
GDH	glutamate dehydrogenase
NBT	nitroblue tetrazolium
2-OG	2-oxoglutarate
2-OGDH	2-oxoglutarate dehydrogenase
PMS	phenazine methosulfate
TCA	cycle, tricarboxylic acid cycle
