# Supplementary material for: Biochemical and Epigenetic Regulation of Glutamate Metabolism in Maize (Zea mays L.) Leaves under Salt Stress
Source: Plants (Basel). 2024 Sep 21;13(18):2651. doi: 10.3390/plants13182651 (PMC11434742; doi:10.3390/plants13182651)
Supplement: Supplementary file 1 [file plants-13-02651-s001.zip › plants-3134950-supplementary.pdf]

Supplementary Table S1. Primers to the genes encoding glutamate dehydrogenase

Gene	Primer	5'-oligonucleotide-3'	Annealing temperature, °C
Gdh1	Forward	GCGGAGAACAAGGGGATCAA	58
	Reverse	ACAGGATCTCGTCTGCCTCT	
Gdh2	Forward	TGATCCAGAGGCAGACGAGA	58
	Reverse	GTAATGCGCGGTCAATGGTC	

Supplementary Table S2. Primers to the genes of glutamate decarboxylase

Gene	Primer	5'-oligonucleotide-3'	Annealing temperature, °C
Gad	Forward	CCGGATCTGTGGTCGTCA	58
	Reverse	CACTACTCCATTAGTACCAGCG	

Supplementary Table S3. Primers to the genes of 2-oxoglutarate dehydrogenase

Gene	Primer	5'-oligonucleotide-3'	Annealing temperature, °C
Ogdh1	Forward	ATTCCAATGACCGTGACAGG	59
	Reverse	AAAAATCGGCGCATCCAATG	
Ogdh3	Forward	GAAGCCATGACTACTCTGCC	61
	Reverse	GCTCCGCATCTTGGTTCATA	

Supplementary Table S4. Primers to the genes encoding Ef-1α

Gene	Primer	5'-oligonucleotide-3'	Annealing temperature, ºC
Ef-1α	Forward	ACCACTGGTGGTTTTGAGGCTGG	58-61
	Reverse	GTTGGTGGACCTCTCAATCATG	

Supplementary Table S5. Oligonucleotides for methyl-specific PCR for the primers of *Gdh1* and *Gdh2*

Gene	M or U	Distance		5'-oligonucleotide-3'	Annealing temperature, ºC
Gdh1	Forward M	1	−490	ATTTGTAGATTTAATCGGTTGGGT	52-53
	Reverse M			AAAAACTAAAATCACTATTCTCGTT	
	Forward U			ATTTGTAGATTTAATTGGTTGGGT	
	Reverse U			AAAAACTAAAATCACTATTCTCATT	
	Forward M	2	−782	TCGTAGTAATTTTTGTTTTGTGG	52-53
	Reverse M			AAAAACTAAAATCACTATTCTCGTT	
	Forward U			TTGTAGTAATTTTTGTTTTGTGG	
	Reverse U			AAAAACTAAAATCACTATTCTCATT	
	Forward M	3	−861	TAAGATTGTATATTTTCGGTGGTAT	52-53
	Reverse M			AAAAACTAAAATCACTATTCTCGTT	
	Forward U			TAAGATTGTATATTTTTGGTGGTAT	
	Reverse U			AAAAACTAAAATCACTATTCTCATT	
Gdh2	Forward M	1	−694	AGATAAGTTAGTTATGGGATGGGC	53
	Reverse M			TACGTCTTCTTAATAACCAAACGAA	
	Forward U			AGATAAGTTAGTTATGGGATGGGTG	
	Reverse U			TACATCTTCTTAATAACCAAACAAA	
	Forward M	2	−735	GGTAAGTGGACGGAAAAGGA	53
	Reverse M			TACGTCTTCTTAATAACCAAACGAA	
	Forward U			GGTAAGTGGATGGAAAAGGA	
	Reverse U			TACATCTTCTTAATAACCAAACAAA	
	Forward M	3	−813	GGTTCGGTTTAGTTTTGAAATAAT	53
	Reverse M			TACGTCTTCTTAATAACCAAACGAA	
	Forward U			GGTTCGGTTTAGTTTTGAAATAAT	
	Reverse U			TACATCTTCTTAATAACCAAACAAA	

Supplementary Table S6. Oligonucleotides for methyl-specific PCR for the primers of *Gad*

Gene	M or U	Distance		5'-oligonucleotide-3'	Annealing temperature, °C
Gad	Forward M	1	−720	TTAAATATTTGAATATCGATTG	53
	Reverse M			TAATAATACTCTAATAATAAAAAAC	
	Forward U			TTAAATATTTGAATATTGATTG	
	Reverse U			TAATAATACTCTAATAATAAAAAAC	
	Forward M	2	−440	TAGGTTGTAGCGATAATA	53
	Reverse M			TAATAATACTCTAATAATAAAAAAC	
	Forward U			TAGGTTGTAGTGATAATA	
	Reverse U			TAATAATACTCTAATAATAAAAAAC	
	Forward M	3	−182	CGAGTATTAGTAAATAATGA	53
	Reverse M			TAATAATACTCTAATAATAAAAAAC	
	Forward U			TGAGTATTAGTAAATAATGA	
	Reverse U			TAATAATACTCTAATAATAAAAAAC	

Supplementary Table S7. Oligonucleotides for methyl-specific PCR for the primers of *Ogdh1* and *Ogdh3*

Gene	Distance		M or U	5'-oligonucleotide-3'	Annealing temperature, ºC
Ogdh1	I	−476	Forward M	GTTTTATATTGTAAAAATTGATCGA	52
			Reverse M	AATTTTCCCATCTAATTATCTCCGT	
			Forward U	GTTTTATATTGTAAAAATTGATTGA	52
			Reverse U	AATTTTCCCATCTAATTATCTCCAT	
	II	−525	Forward M	GTTTAGATTGGTTATGCGT	52
			Reverse M	AATTTTCCCATCTAATTATCTCCGT	
			Forward U	GTTTAGATTGGTTATGTGT	52
			Reverse U	AATTTTCCCATCTAATTATCTCCAT	
	III	−646	Forward M	TTTTTTGGATGATAGTTTTGGCGT	52
			Reverse M	AATTTTCCCATCTAATTATCTCCGT	
			Forward U	TTTTTTGGATGATAGTTTTGGTGT	52
			Reverse U	AATTTTCCCATCTAATTATCTCCAT	
Ogdh3	I	−300	Forward M	TGTTATGGGAAGAAATCGC	51
			Reverse M	CCCTACACAAAAACAAAAA	
			Forward U	TGTTATGGGAAGAAATTGC	51
			Reverse U	CCCTGCACAAAAACAAAAA	
	II	−366	Forward M	TTTTAGTGTTTTGTACGG	51
			Reverse M	CCCTACACAAAAACAAAAA	
			Forward U	TTTTAGTGTTTTGTATGG	51
			Reverse U	CCCTGCACAAAAACAAAAA	
	III	−387	Forward M	GTGCGTAATTGTTCGA	49
			Reverse M	CCCTACACAAAAACAAAAA	
			Forward U	GTGTGTAATTGTTTGA	49
			Reverse U	CCCTGCACAAAAACAAAAA	

Note: The minus sign indicates at what distance from the beginning of the first exon of the gene the studied cytosine is located; I, II, III - different groups of primers. Cytosine in each group of primers differed by the presence of methylation (M) or its absence (U).

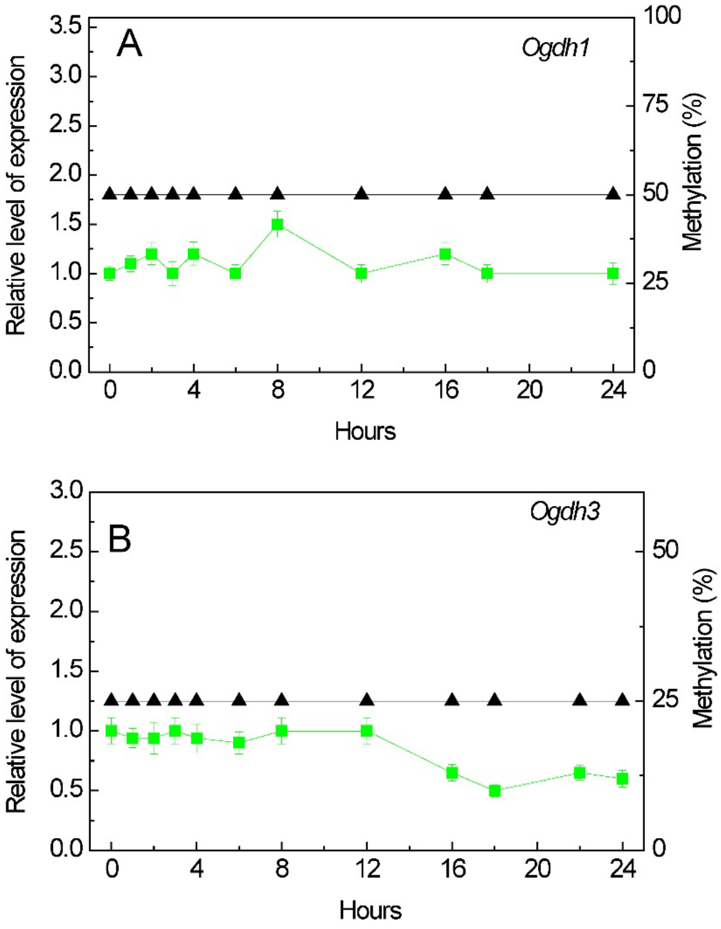

Supplementary Figure S1. Changes in the relative levels of transcripts of the genes *Ogdh1* (A) and *Ogdh3* (B) (green squares) and of the fraction of methylation of their promoters (black triangles) in the control (non-stressed) maize plants.

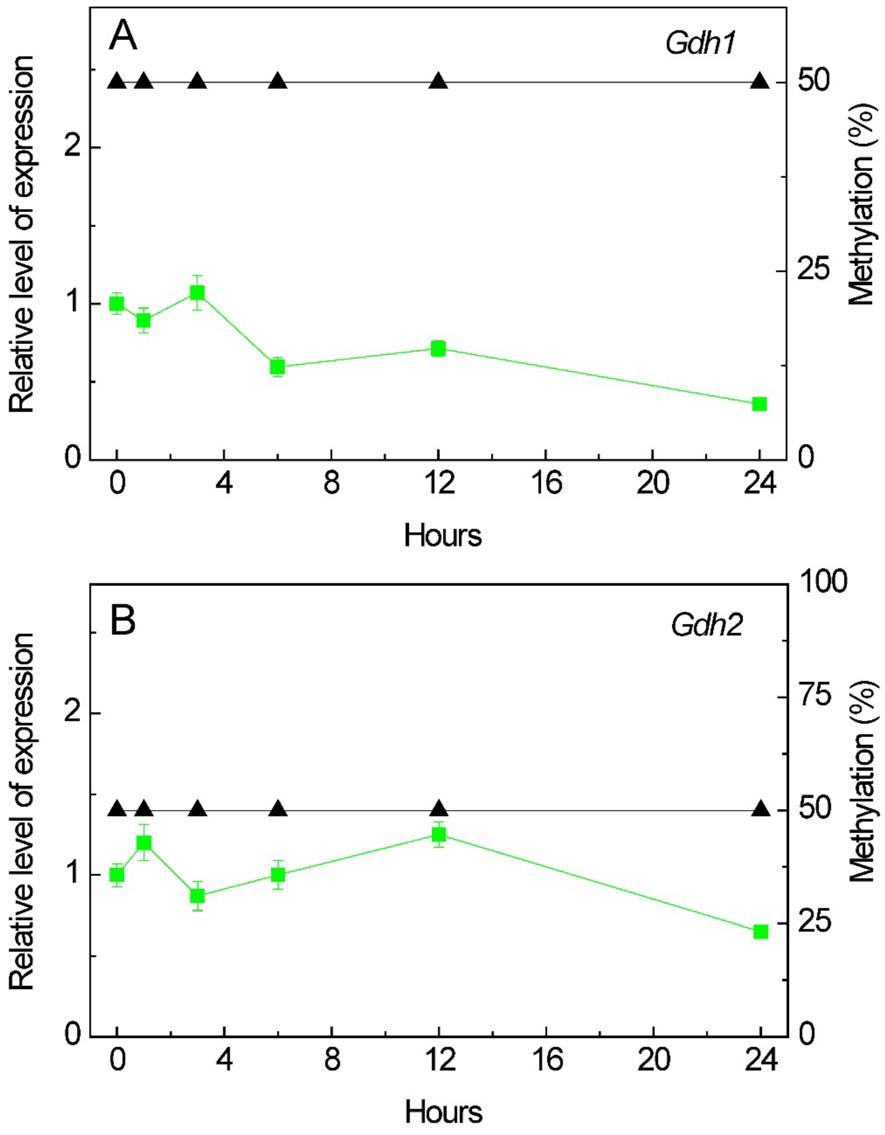

Supplementary Figure S2. Changes in the relative levels of transcripts of the genes *Gdh1* (A) and *Gdh2* (B) (green squares) and of the fraction of methylation of their promoters (black triangles) in the control (non-stressed) maize plants.

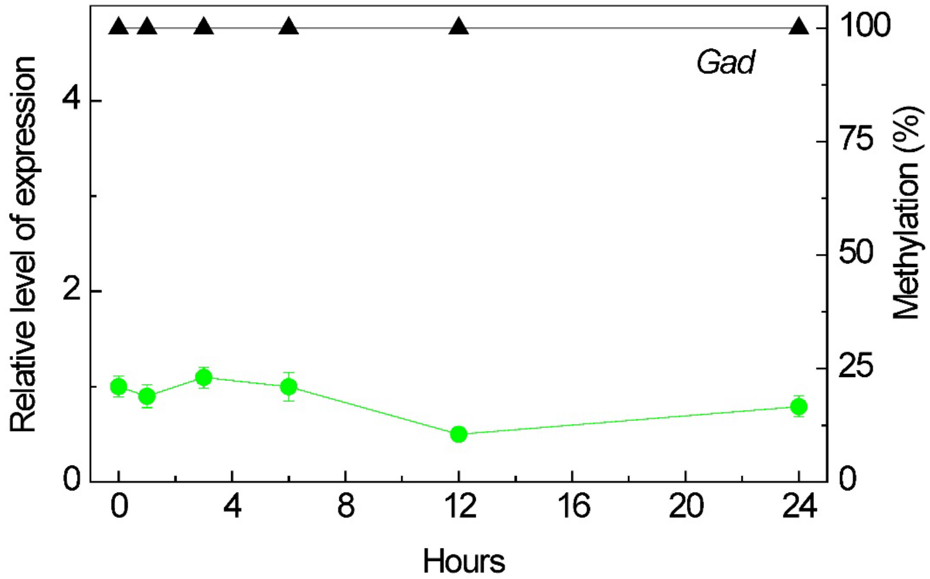

Supplementary Figure S3. Changes in the relative levels of transcripts of the gene *Gad* (green squares) and of the fraction of methylation of its promoters (black triangles) in the control (non-stressed) maize plants.
